# Research on the Species Diversity and Distribution Patterns of Wild *Ribes* in Northeast Asia

**DOI:** 10.3390/plants14121780

**Published:** 2025-06-11

**Authors:** Ximing Zhao, Dandan Zhao, Xinrui Ge, Yin Zhang, Yuxiao Du, Jingguo Liu, Yuning Liu, Hongfeng Wang, Baojiang Zheng

**Affiliations:** 1 College of Life Sciences, Northeast Forestry University, Harbin 150040, China; xiaomingfancy@163.com (X.Z.); fina370@163.com (D.Z.); gexinrui23@163.com (X.G.); 15329196730@163.com (Y.Z.); duyuxiao1999@163.com (Y.D.); 18846779641@163.com (J.L.); lyning616@163.com (Y.L.); 2 College of Forestry, Northeast Forestry University, Harbin 150040, China; 3 Northeast Asia Biodiversity Research Center, Harbin 150040, China

**Keywords:** Northeast Asia, *Ribes*, species diversity, horizontal distribution, vertical distribution

## Abstract

*Ribes* is the only genus in the Grossulariaceae family and holds considerable economic importance. Northeast Asia represents one of the major global centers of *Ribes* distribution. This study presents the first comprehensive investigation focused on this region, examining the diversity, distribution patterns, and environmental determinants of wild *Ribes* species through field surveys and a review of the relevant literature. Results indicate the presence of 36 species (including 8 varieties) from 7 subgenera of wild *Ribes* across Northeast Asia, predominantly belonging to the subgenera *Berisia*, *Ribes*, and *Grossularia*. The species are unevenly distributed throughout the region: (1) The Russian Far East exhibits the highest species richness, with 21 species from 5 subgenera, followed by Northeast China (16 species, 6 subgenera), Japan (12 species, 7 subgenera), Mongolia (10 species, 3 subgenera), South Korea (9 species, 5 subgenera), and North Korea (8 species, 5 subgenera). These findings suggest that the Russian Far East currently serves as the core distribution center for *Ribes* in Northeast Asia. (2) The species diversity of wild *Ribes* exhibits a unimodal latitudinal pattern, peaking between 47° N and 52° N. (3) A similar unimodal trend is evident along altitudinal gradients, with most species occurring between 500 m and 1500 m. (4) Species richness is primarily influenced by temperature stability and extreme low temperatures, followed by precipitation seasonality and elevation, while annual precipitation shows a relatively minor effect. This study offers crucial baseline data for the conservation and sustainable utilization of *Ribes* in Northeast Asia.

## 1. Introduction

*Ribes*, the sole genus in the Grossulariaceae family, includes approximately 200 species globally, primarily distributed in East Asia, North America, and the Andes Mountains of South America [1]. At the continental scale, Asia harbors the highest species richness (over 80 taxa), with major concentrations in East Asia and northern Asia (extending to Siberia and the Russian Far East), and a limited representation in South Asia, mainly in northern India and the Himalayas. In contrast, distributions in Southwest Asia are sparse—confined to Afghanistan, Iran, and Lebanon—while Southeast Asia is nearly devoid of *Ribes*. North America sustains over 70 species, ranging latitudinally from Alaska to Mexico, with significant clustering along the Pacific coast. South America hosts 38 species, predominantly in the Andean cordillera [1]. According to Takhtajan’s division of the world flora, East Asia contains the largest number of *Ribes* species (44 species), followed by the circumboreal region with 34 species, and the Madrean region with 30 species [2].

The primary chemical constituents of *Ribes* plants include phenolic glycosides, flavonoids, procyanidins, and polysaccharides, which endow them with high nutritional value [3,4]. Many traditional uses of *Ribes* are associated with its biological activities, and modern pharmacological studies have substantiated these applications. Research has demonstrated that both extracts and purified compounds exhibit significant antioxidant, anti-inflammatory, anticancer, neuroprotective, anti-obesity, and therapeutic effects on urinary and cardiovascular diseases [5,6,7,8,9]. In addition, studies have shown that *Ribes* extracts can serve as reducing agents in the synthesis of nanomaterials [10,11,12,13]. In summary, *Ribes* represents an economically valuable plant group with both edible and medicinal properties and promising development prospects.

Currently, there is no comprehensive report on the species diversity and distribution patterns of wild *Ribes* in Northeast Asia. Based on long-term sampling and line and road encounter surveys conducted by the research team—combined with recent advances in regional studies and data from major biodiversity platforms for bioinformatics analysis—this study examines wild *Ribes* in Northeast Asia from multiple perspectives. These include assessments of species diversity, the distribution of taxa which are present in more than one geographic unit and taxa present in one geographic unit only, and both horizontal and vertical distribution patterns, with the aim of providing a theoretical basis for biodiversity research in Northeast Asia and offering references for the conservation and utilization of *Ribes* plants.

## 2. Results

### 2.1. Species Diversity of Wild Ribes in Northeast Asia

#### 2.1.1. Wild *Ribes* Species Composition

In Northeast Asia, there are 7 subgenera and a total of 36 species (including 8 varieties) of wild *Ribes* (Table 1), which account for 53.85% of the subgenera and 18% of the species of this genus, respectively. Among them, there are 11 species (including 4 varieties) in the subg. *Berisia*, 11 species (including 2 varieties) in the subg. *Ribesia*, 6 species in the subg. *Coreosma*, 4 species (including 1 variety) in the subg. *Grossularia*, 2 species (including 1 variety) in the subg. *Hemibotrya*, 1 species in the subg. *Grossularioides*, and 1 species in subg. *Hertiera* [14,15,16,17,18,19,20,21,22,23,24,25,26,27,28].

#### 2.1.2. Analysis of Wild *Ribes* Species Composition in Each Country and Region of Northeast Asia

**Figure 1:**
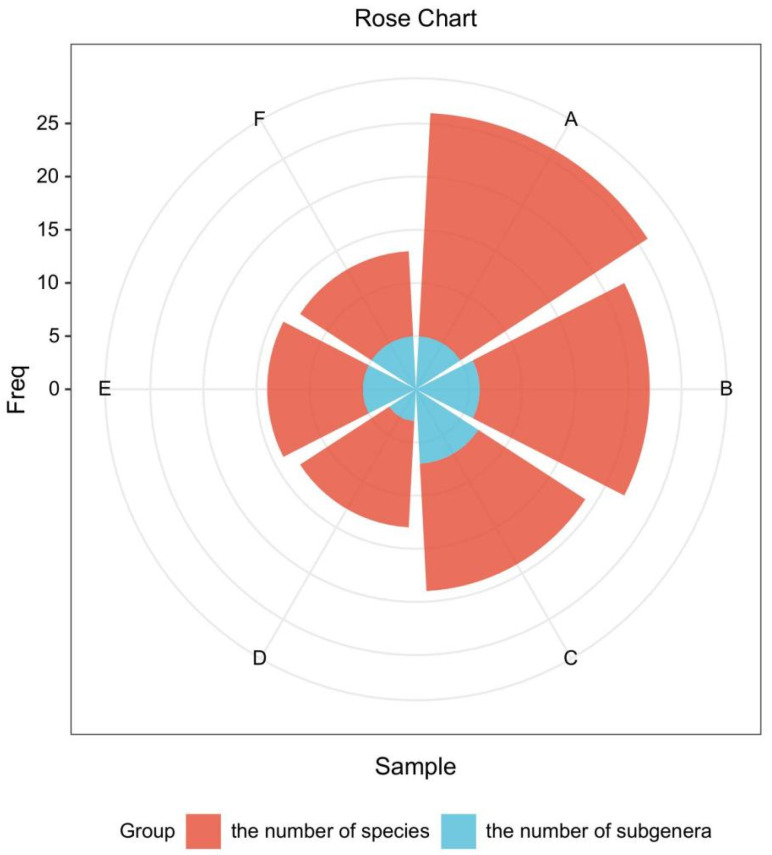
Distribution of the number of species and subgenera of wild *Ribes* in various countries and regions of Northeast Asia. A: Northeast Asia region of Russia; B: Northeast China; C: Japan; D: Mongolia; E: South Korea; F: North Korea.

According to field surveys and data analysis, the Northeast Asia region of Russia exhibits the highest species richness, encompassing 5 subgenera and a total of 21 species (Table 2). Northeast China harbors 6 subgenera and 16 species, while Japan is home to 7 subgenera and 12 species. In Mongolia, 3 subgenera and 10 species are recorded, and South Korea contains 5 subgenera and 9 species. North Korea has the fewest, with 5 subgenera and 8 species (Figure 1).

Based on field surveys and data analysis, no common species are distributed across all six countries and regions (Table 3). However, the Northeast Asia region of Russia, Northeast China, Japan, South Korea, and North Korea share the highest number of taxa, with a total of four. The concentration of these shared taxa in these five regions likely reflects a complex interplay of factors, including large-scale influences like geographical proximity and historical dispersal routes, as well as fine-scale environmental conditions such as the availability of suitable ecological niches, favorable mesoclimates, and specific microclimates.

The highest number of endemic species is found in the Northeast Asia region of Russia and Japan, each harboring four species. The taxa present in one geographic unit only in Russia are *Ribes dikuscha* Fisch. ex Turcz., *Ribes fontaneum* Bochkarn., *Ribes pallidiflorum* Pojark., and *Ribes saxatile* Pall. In Japan, the endemic species include *Ribes ambiguum* Maxim., *Ribes fujisanense* S. Sakag. & M. Oishi, *Ribes japonicum* Maxim., and *Ribes sinanense* F. Maek. Northeast China ranks second in endemic species, with two: *Ribes fasciculatum* var. *chinense* Maxim. and *Ribes giraldii* Jancz. Mongolia has one endemic species, *Ribes heterotrichum* C.A. Mey. In contrast, no endemic species are found in South Korea or North Korea.

**Figure 2:**
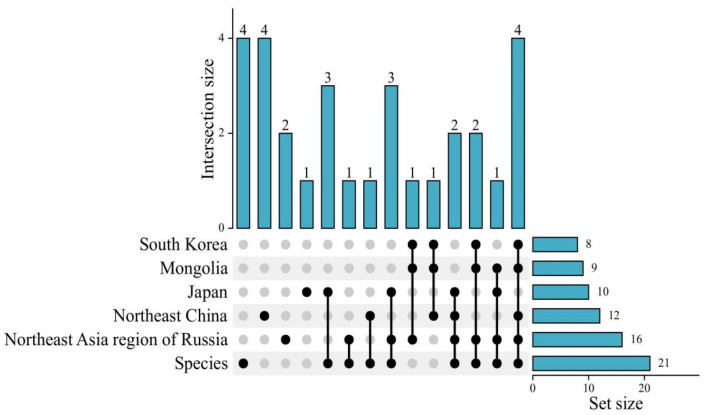
The number of common and endemic species in various floristic regions of Northeast Asia. The dot matrix at the bottom indicates combinations of countries/regions. Intersection size represents the number of species found exclusively in that specific combination of regions. Set size displays the total number of species within each individual country/region.

The low number of endemic species in Mongolia may be partly explained by its shared borders with Northeast China and the Northeast Asia region of Russia, which facilitates taxa exchange, while Changbai Mountain, a relevant biogeographical zone, is shared between Northeast China and the Korean Peninsula, resulting in a common flora (Figure 2).

#### 2.1.3. Distribution Center of *Ribes* in Northeast Asia

To accurately determine the distribution center of wild *Ribes* in Northeast Asia, this study considers three equally weighted dimensions: species count, subgenus count, and number of endemic species. These factors were normalized for comparative analysis.

**Figure 3:**
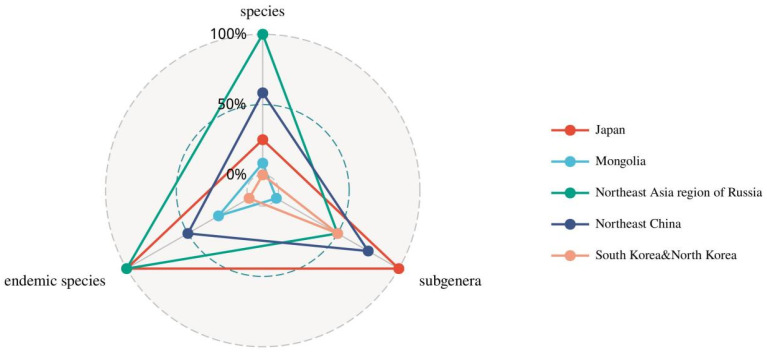
Distribution of three dimensions of countries and regions in Northeast Asia on a radar chart.

Based on the radar chart, the ranking of distribution areas from largest to smallest is as follows: the Northeast Asia region of Russia, Japan, Northeast China, Mongolia, South Korea, and North Korea. Notably, the distribution patterns of North Korea and South Korea are identical on the radar chart. The Northeast Asia region of Russia occupies the largest area, indicating that it serves as the primary distribution center for wild *Ribes* in Northeast Asia (Figure 3).

This result is closely linked to the region’s vast landmass and complex topography. Its extensive territory provides a broad latitudinal range, while the diverse mountainous terrain creates significant altitudinal variation. These factors collectively offer *Ribes* taxa ample horizontal and vertical distribution space, facilitating their rich biodiversity in this region.

### 2.2. Distribution Patterns of Ribes in Northeast Asia and Their Correlation with Environmental Factors

#### 2.2.1. Spatial Distribution Pattern of Wild *Ribes* Species Richness in Northeast Asia

**Figure 4:**
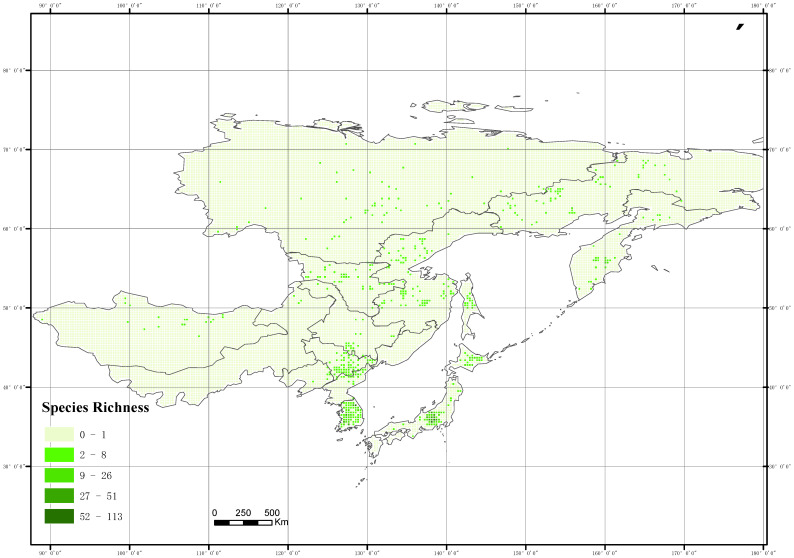
Species distribution pattern of *Ribes* L. in Northeast Asia.

The spatial distribution of wild *Ribes* species richness across Northeast Asia is highly heterogeneous, with the number of taxa per grid cell ranging from 0 to 141 (Figure 4).

Horizontally, wild *Ribes* taxa are distributed between 32° N and 72° N, with diversity hotspots concentrated between 47° N and 52° N. Vertically, their distribution ranges from sea level to 3147 m.

A distinct latitudinal gradient is observed: from 32° N to 37° N, only 10 taxa are recorded, indicating relatively low diversity. Species richness increases with latitude, peaking at 47° N–52° N with 20 taxa, and subsequently declines toward higher latitudes, reaching a minimum of 4 taxa at 67° N–72° N. This unimodal pattern is likely influenced by decreasing temperatures at higher latitudes, which constrain the survival of cold-sensitive taxa.

**Figure 5:**
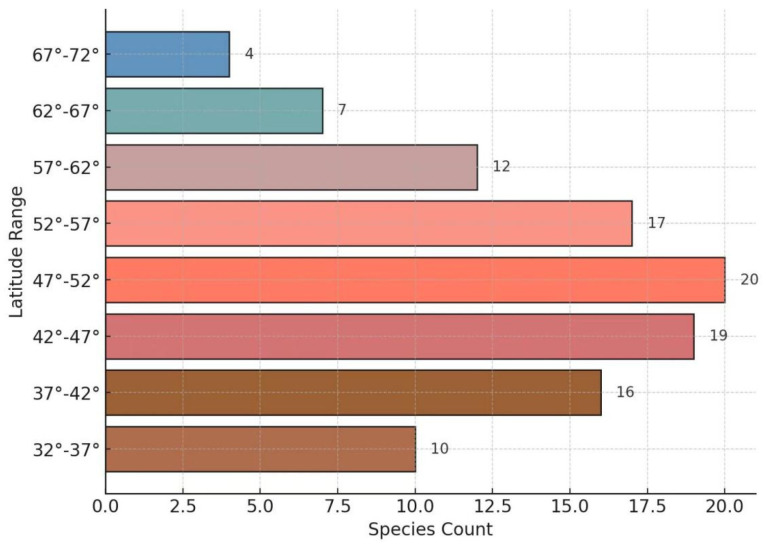
Distribution of *Ribes* in Northeast Asia across various latitude ranges.

Among all taxa, *Ribes triste* Pall. exhibits the broadest latitudinal range (32.19°), followed by *Ribes nigrum* L.(28.82°) (Figure 5).

**Figure 6:**
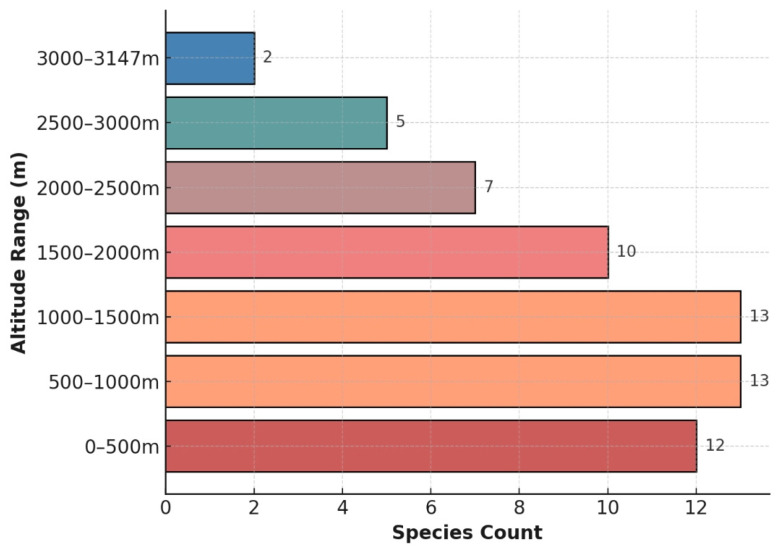
Vertical distribution patterns of wild *Ribes* L. in Northeast Asia.

Vertically, wild *Ribes* taxa occur from 0 to 3147 m. Within the 0–1500 m range, species richness increases with elevation, with the highest diversity observed in the 500–1000 m and 1000–1500 m ranges, each supporting 13 taxa. Above 1500 m, richness declines with elevation, likely due to decreasing temperatures limiting the presence of less cold-adapted taxa. At elevations above 3000 m, richness reaches its lowest point, with only 2 taxa recorded between 3000 m and 3667 m (Figure 6). This pattern is consistent with general ecological trends where biodiversity decreases with increasing elevation due to harsher climatic conditions.

#### 2.2.2. Relationship Between Wild *Ribes* Species Richness Patterns and Environmental Factors in Northeast Asia

This study investigated the relationship between various environmental variables and the species richness of wild *Ribes* in Northeast Asia (Table 4). The results indicated that temperature-related climatic factors exert the most substantial influence on species richness. Among these, isothermality (BIO3; mean ± SD: 23.246 ± 5.584) exhibited the strongest positive correlation with species richness (T = 13.59, *p* < 0.001). In addition, both annual mean temperature (BIO1; −1.906 ± 7.515 °C) and mean temperature of the coldest quarter (BIO11; −18.707 ± 10.512 °C) were highly significant predictors (*p* < 0.001), emphasizing the limiting effect of extreme cold on species distribution.

Furthermore, the mean temperature of the wettest quarter (BIO8; 13.837 ± 5.506 °C) and mean temperature of the warmest quarter (BIO10; 14.643 ± 5.192 °C) were also significantly correlated with species richness (*p* < 0.001), highlighting the ecological importance of growing-season thermal conditions.

Among precipitation-related variables, annual precipitation (BIO12; 469.173 ± 275.078 mm) and precipitation of the driest month (BIO14; 13.942 ± 12.228 mm) were not significantly associated with species richness (*p* > 0.05). In contrast, precipitation seasonality (BIO15; 62.705 ± 29.082) showed a strong positive correlation (*p* < 0.001), indicating that the temporal variability of precipitation exerts greater ecological influence on *Ribes* distribution than total annual precipitation.

Regarding topographic factors, mean elevation (ELE; 683.056 ± 974.05 m) was significantly correlated with species richness (*p* = 0.008), underscoring the role of terrain and elevation gradients.

In summary, the distribution of *Ribes* species richness in Northeast Asia is primarily shaped by temperature stability and tolerance to extreme cold, followed by precipitation seasonality and topography. In contrast, total annual precipitation has a relatively minor effect.

## 3. Discussion

### 3.1. Species Diversity and Spatial Distribution Patterns of Wild Ribes Species in Northeast Asia

The genus *Ribes* includes 13 subgenera globally [1,29], 5 of which are unique to North America and 1 to South America. In Northeast Asia, 7 subgenera are represented, encompassing 28 species and 8 varieties. While this region does not host the highest number of *Ribes* species in Asia, it exhibits the greatest subgeneric diversity on the continent. Across Asia as a whole, the genus includes approximately 80 species within the same 7 subgenera.

Among the seven subgenera occurring in Asia, three—*Berisia*, *Ribesia*, and *Grossularia*—contain more than twenty species each. In Northeast Asia, three subgenera exceed five species: *Berisia*, *Ribesia*, and *Coreosma*. Notably, *Grossularia* is represented by only three species in this region and is entirely absent from the Russian portion of Northeast Asia.

Among the six countries and regions within Northeast Asia, Japan displays the highest subgeneric diversity, harboring one more subgenus than Northeast China—*Hertiera*. This subgenus includes six known species: *Ribes erythrocarpum* Cov. & Leib, *Ribes glandulosum* Grauer, *Ribes howellii* Greene, *Ribes laxiflorum* Pursh, *Ribes prostratum* L’Hér., and *Ribes sachalinense* (F. Schmidt) Nakai. The group is disjunctively distributed in North America and East Asia [1]. Of these, five are widespread in North America, while only *R. sachalinense* occurs in East Asia, specifically in northern Japan and on Sakhalin Island, Russia. Its distribution is primarily restricted to alpine zones of northern islands in Northeast Asia.

With respect to latitude, *Ribes* species richness exhibits a unimodal distribution, peaking in the mid-latitudes (47–52° N) with 20 species, and declining toward both lower (32–37° N, 10 species) and higher latitudes (67–72° N, 4 species). This trend may reflect the limiting effects of temperature: cold stress in high-latitude zones allows only cold-tolerant species (e.g., *Ribes triste* Pall. and *Ribes nigrum* L.) to survive, while high summer temperatures or competitive exclusion may constrain diversity in low-latitude regions.

Along elevational gradients, species richness is highest between 500 and 1500 m, with 13 species recorded. Only two species persist above 3000 m, a pattern consistent with the “mid-domain peak model” and likely driven by combined effects of low temperatures and reduced oxygen availability at higher altitudes.

### 3.2. Temperature as the Primary Driver of Ribes Species Richness

Understanding the spatial distribution patterns of species diversity and their relationship with environmental factors not only helps to better understand the mechanisms behind species diversity distribution but also provides scientific evidence for developing effective biodiversity conservation strategies [30]. Climatic analyses indicate that among temperature, precipitation, and topographic variables, temperature-related factors have the greatest influence on *Ribes* species richness in Northeast Asia.

In particular, isothermality (BIO3) shows a strong positive correlation with species richness (*p* < 0.001), suggesting that stable thermal regimes favor broader species distributions. Conversely, annual mean temperature (BIO1) and mean temperature of the coldest quarter (BIO11) show significant negative correlations, implying that extreme cold and reduced temperature stability constrain *Ribes* distribution.

Overall, the species richness of wild *Ribes* in Northeast Asia is influenced by multiple factors. Given the geographic and numerical variation in species distribution across the region, future research should focus on more targeted analyses of specific taxonomic groups within defined subregions. This includes examining whether additional factors—such as historical climate dynamics, human activities, and soil conditions—may also affect the distribution of wild *Ribes* species. A deeper understanding of these influences would contribute to more informed and effective conservation strategies for *Ribes* in Northeast Asia.

## 4. Materials and Methods

### 4.1. Research Area

Northeast Asia includes Northeastern China, the Russian Far East and Siberia, Japan, South Korea, Mongolia, and North Korea. In this study, the two federal districts of Russia are collectively referred to as the Russian Northeast Asia region.

Northeastern China consists of three northeastern provinces and the eastern part of Inner Mongolia, including Xilingol League, Chifeng City, Tongliao City, Hinggan League, and Hulunbuir City. According to official information from the Far Eastern Federal District Government of Russia (http://www.dfo.gov.ru/district/, accessed on 6 April 2024), the Russian Far East encompasses Amurskaya Oblast, Jewish Autonomous Oblast, Kamchatsky Krai, Magadanskaya Oblast, Primorsky Krai, the Sakha (Yakutia) Republic, Sakhalin Oblast, Khabarovsk Krai, Chukotka Autonomous Okrug, the Republic of Buryatia, and Zabaykalsky Krai. The Siberian region of Russia, as included in this study, consists of the Altai Republic, Altai Krai, Irkutsk Oblast, Kemerovo Oblast, Krasnoyarsk Krai, Novosibirsk Oblast, Omsk Oblast, Tomsk Oblast, the Tyva Republic, and the Republic of Khakassia.

This region features four major climate types: temperate continental climate (including subarctic coniferous forests), polar climate (tundra and ice sheet), temperate monsoon climate, and subtropical monsoon climate with humid monsoon influences.

### 4.2. Data Collection

#### 4.2.1. Species Distribution Data

During the biodiversity survey of wild *Ribes* taxa in Northeast China, our research team conducted transect-based investigations supplemented by quadrat sampling, taking into account the biological and ecological characteristics of *Ribes* plants as well as the distribution patterns of different taxa.

Between 2012 and 2023, our research team conducted multiple field investigations targeting *Ribes* plant resources across Northeast China. These included three expeditions to the Daxing’an Mountains, two to the Xiaoxing’an Mountains, Zhangguangcai Mountains, Laoye Mountains, and Wanda Mountains in Heilongjiang Province, two to the Changbai Mountains in Jilin Province, two to the eastern region of Liaoning Province and the Liaodong Peninsula, and one to the western and northern regions of Liaoning Province. This survey was conducted along multiple transect lines: 8 in the Daxing’an Mountains, 6 in the Xiaoxing’an Mountains, 15 in the Changbai Mountains, and 5 in the Liaodong Peninsula. The length of each transect ranged from 1.5 to 3 km.

Special emphasis was placed on the vertical distribution of *Ribes* across distinct natural geographical units (e.g., the Daxing’an, Xiaoxing’an, and Changbai Mountains), through which we gathered extensive data including distribution records, photographs, and specimen collections.

Survey routes were typically established in areas with significant topographic variation, diverse vegetation types, and vigorous plant growth. These routes were designed to cover gradients ranging from valleys to ridges, low to high elevations, sunny to shady slopes, and downstream to upstream sections of rivers.

For each target taxa encountered, we recorded its location (longitude and latitude), topography, elevation, and soil conditions. Specimens with uncertain identification were promptly photographed and collected for further analysis.

For rare *Ribes* taxa found in the survey area, quadrat sampling was conducted using 5 m × 5 m (25 m^2^) plots. Within each quadrat, we documented the number of individuals, plant height, basal diameter, and associated taxa. This approach ensured comprehensive data collection on the distribution and ecological preferences of *Ribes* taxa in the region.

These efforts aimed at providing a comprehensive understanding of the species composition, spatial distribution, and ecological conditions of *Ribes* in Northeast China. The resulting distribution data are compiled and summarized in Table S1.

To compile a regional species list of wild *Ribes* across the rest of Northeast Asia, various floristic references were consulted.

Additionally, online botanical databases such as Flora of China (https://www.iplant.cn/), the International Plant Name Index (IPNI, https://www.ipni.org/), and Plants of the World Online (POWO, https://powo.science.kew.org/) were used for taxonomic verification. Species diversity data from other countries and regions were acquired using rectangular selection and specimen query tools provided by the Global Biodiversity Information Facility (GBIF) [31].

The final dataset includes 36 species and infraspecific taxa representing 7 subgenera of wild *Ribes* in Northeast Asia, totaling 8224 distribution records (Table S2).

#### 4.2.2. Environmental Data

Two primary datasets were used in this study, climate data and topographic data, both derived from the WorldClim 2.1 database (https://www.worldclim.org). The climate dataset includes 19 bioclimatic variables (BIO1–BIO19) related to temperature and precipitation, representing averages from 1970 to 2000. Topographic information was obtained from SRTM elevation data within the same platform.

### 4.3. Data Processing

#### 4.3.1. Species Distribution and Richness Calculation

Species richness was defined as the total number of *Ribes* species occurring within each grid cell. First, an equal-area conic projection was applied using ArcGIS (version 10.8). To ensure uniform analytical units, the Asian administrative boundary map was then converted into 1 km × 1 km equal-area grid cells using a fishnet tool, and the central coordinate of each grid was extracted. Based on these coordinates, species richness values were assigned to each grid cell. The Jenks natural breaks classification method in ArcGIS was subsequently applied to divide species richness into five levels.

GBIF records were initially filtered and organized using Excel, followed by the compilation of horizontal and vertical distribution patterns of *Ribes* in Northeast Asia. Data visualization, including bar charts, radar plots, and chord diagrams, was conducted using bioinformatics tools such as Hiplot (version Pro 2.0).

With the aim of maintaining scientific rigor, the following data processing principles were observed:

- For analyses across the entire Northeast Asian region, both species and infraspecific taxa (e.g., varieties) were treated as separate species.
- In analyses at the country or region level, if both a species and its variety co-occurred in the same area, the variety was merged under the original species.

#### 4.3.2. Correlation Analysis

With the aim of normalizing skewed data distributions, logarithmic transformations Y = log(Y + 1) were applied to certain variables. Species richness was treated as the dependent variable, and multiple linear regression (MLR) was used to evaluate the influence of various environmental factors. The significance of regression coefficients (β) was assessed via t-tests, and corresponding T-values and *p*-values were calculated. Model robustness was ensured by testing for multicollinearity using the variance inflation factor (VIF), with VIF < 5 considered acceptable.

All statistical analyses were performed using RStudio (version 4.5.0).

## 5. Conclusions

This study provides a comprehensive assessment of the wild *Ribes* species in Northeast Asia. By integrating publicly available data from the Global Biodiversity Information Facility (GBIF), we analyzed species diversity along with the horizontal and vertical distribution patterns of wild *Ribes* in the region. Compared to previous studies, this research incorporates the latest advances in subgeneric classification within the genus *Ribes*.

The results reveal that Northeast Asia hosts 36 species, including 8 varieties, across 7 subgenera, with *Berisia*, *Ribesia*, and *Coreosma* being the dominant groups. The current distribution center of *Ribes* in this region is located in the Russian Far East. Species richness displays a unimodal pattern along both latitudinal and elevational gradients: the highest diversity occurs between 47° N and 52° N (20 species), and within an elevation range of 500–1500 m (13 species).

Among environmental variables, temperature-related factors—particularly isothermality and extreme low temperatures—are identified as the primary drivers of *Ribes* species richness in Northeast Asia.

Wild *Ribes* in this region is characterized by high species diversity, broad geographic distribution, and distinct spatial gradients, suggesting strong potential for further ecological study and applied research. Future work should focus on exploring its economic value and ecological applications, as well as enhancing conservation efforts for this diverse genus.

## Figures and Tables

**Table 1:** Subgenera and species of wild *Ribes* in Northeast Asia.

Subgenera	Scientific Name
subg. *Berisia*	*Ribes diacantha* Pall.
	*Ribes dikuscha* Fisch. ex Turcz.
	*Ribes giraldii* var. *cuneatum* F.T.Wang & S.X.Li
	*Ribes giraldii* var. *polyanthum* Kitag.
	*Ribes heterotrichum* C.A.Mey.
	*Ribes komarovii* Pojark.
	*Ribes komarovii* var. *cuneifolium* Liou
	*Ribes maximowiczianum* Kom.
	*Ribes pulchellum* Turcz.
	*Ribes pulchellum* var. *manshuriense* F.T.Wang & S.X.Li
	*Ribes saxatile* Pall.
subg. *Ribesia*	*Ribes altissimum* Turcz. ex Pojark.
	*Ribes ambiguum* Maxim.
	*Ribes atropurpureum* C.A.Mey.
	*Ribes latifolium* Jancz.
	*Ribes mandshuricum* (Maxim.) Kom.
	*Ribes mandshuricum* var. *subglabrum* Kom.
	*Ribes meyeri* Maxim.
	*Ribes palczewskii* (Jancz.) Pojark.
	*Ribes pallidiflorum* Pojark.
	*Ribes triste* Pall.
	*Ribes triste* var. *repens* (A. I. Baranov) L. T. Lu
subg. *Coreosma*	*Ribes fontaneum* Bochkarn.
	*Ribes frangrans* Pallas.
	*Ribes graveolens* Bunge
	*Ribes japonicum* Maxim.
	*Ribes nigrum* L.
	*Ribes procumbens* Pall.
subg. *Grossularia*	*Ribes burejense* F.Schmidt
	*Ribes burejense* var. *villosum* L.T.Lu
	*Ribes fujisanense* S.Sakag. & M.Oishi
	*Ribes sinanense* F.Maek.
subg. *Hemibotrya*	*Ribes fasciculatum* Siebold & Zucc.
	*Ribes fasciculatum* var. *chinense* Maxim.
subg. *Grossularioides*	*Ribes horridum* Rupr. ex Maxim.
subg. *Hertiera*	*Ribes sachalinense* (F.Schmidt) Nakai

**Table 2:** Species of Wild *Ribes* in Northeast Asian countries and regions.

Country/Region	Subgenera	Scientific Name
Northeast Asia region of Russia	subg. *Berisia*	*Ribes diacantha* Pall.
	subg. *Berisia*	*Ribes dikuscha* Fisch. ex Turcz.
	subg. *Berisia*	*Ribes komarovii* Pojark.
	subg. *Berisia*	*Ribes maximowiczianum* Kom.
	subg. *Berisia*	*Ribes pulchellum* Turcz.
	subg. *Berisia*	*Ribes saxatile* Pall.
	subg. *Coreosma*	*Ribes fontaneum* Bochkarn.
	subg. *Coreosma*	*Ribes frangrans* Pallas.
	subg. *Coreosma*	*Ribes graveolens* Bunge
	subg. *Coreosma*	*Ribes nigrum* L.
	subg. *Coreosma*	*Ribes procumbens* Pall.
	subg. *Hertiera*	*Ribes sachalinense* (F.Schmidt) Nakai
	subg. *Ribesia*	*Ribes altissimum* Turcz. ex Pojark.
	subg. *Ribesia*	*Ribes atropurpureum* C.A.Mey.
	subg. *Ribesia*	*Ribes latifolium* Jancz.
	subg. *Ribesia*	*Ribes mandshuricum* (Maxim.) Kom.
	subg. *Ribesia*	*Ribes meyeri* Maxim.
	subg. *Ribesia*	*Ribes palczewskii* (Jancz.) Pojark.
	subg. *Ribesia*	*Ribes pallidiflorum* Pojark.
	subg. *Ribesia*	*Ribes triste* Pall.
	subg. *Grossularioides*	*Ribes horridum* Rupr. ex Maxim.
Northeast China	subg. *Berisia*	*Ribes diacantha* Pall.
	subg. *Berisia*	*Ribes giraldii var. cuneatum* F.T.Wang & S.X.Li
	subg. *Berisia*	*Ribes giraldii* var. *polyanthum* Kitag.
	subg. *Berisia*	*Ribes komarovii* Pojark.
	subg. *Berisia*	*Ribes maximowiczianum* Kom.
	subg. *Berisia*	*Ribes pulchellum* Turcz.
	subg. *Coreosma*	*Ribes frangrans* Pallas.
	subg. *Coreosma*	*Ribes nigrum* L.
	subg. *Coreosma*	*Ribes procumbens* Pall.
	subg. *Grossularia*	*Ribes burejense* F.Schmidt
	subg. *Hemibotrya*	*Ribes fasciculatum* var. *chinense* Maxim.
	subg. *Ribesia*	*Ribes altissimum* Turcz. ex Pojark.
	subg. *Ribesia*	*Ribes latifolium* Jancz.
	subg. *Ribesia*	*Ribes mandshuricum* (Maxim.) Kom.
	subg. *Ribesia*	*Ribes palczewskii* (Jancz.) Pojark.
	subg. *Ribesia*	*Ribes triste* Pall.
	subg. *Grossularioides*	*Ribes horridum* Rupr. ex Maxim.
Japan	subg. *Berisia*	*Ribes maximowiczianum* Kom.
	subg. *Coreosma*	*Ribes japonicum* Maxim.
	subg. *Coreosma*	*Ribes nigrum* L.
	subg. *Coreosma*	*Ribes procumbens* Pall.
	subg. *Grossularia*	*Ribes fujisanense* S.Sakag. & M.Oishi
	subg. *Grossularia*	*Ribes sinanense* F.Maek.
	subg. *Hemibotrya*	*Ribes fasciculatum* Siebold & Zucc.
	subg. *Hertiera*	*Ribes sachalinense* (F.Schmidt) Nakai
	subg. *Ribesia*	*Ribes ambiguum* Maxim.
	subg. *Ribesia*	*Ribes latifolium* Jancz.
	subg. *Ribesia*	*Ribes triste* Pall.
	subg. *Grossularioides*	*Ribes horridum* Rupr. ex Maxim.
Mongolia	subg. *Berisia*	*Ribes diacantha* Pall.
	subg. *Berisia*	*Ribes heterotrichum* C.A.Mey.
	subg. *Berisia*	*Ribes pulchellum* Turcz.
	subg. *Coreosma*	*Ribes frangrans* Pallas.
	subg. *Coreosma*	*Ribes graveolens* Bunge
	subg. *Coreosma*	*Ribes nigrum* L.
	subg. *Coreosma*	*Ribes procumbens* Pall.
	subg. *Ribesia*	*Ribes altissimum* Turcz. ex Pojark.
	subg. *Ribesia*	*Ribes atropurpureum* C.A.Mey.
	subg. *Ribesia*	*Ribes meyeri* Maxim.
South Korea	subg. *Berisia*	*Ribes diacantha* Pall.
	subg. *Berisia*	*Ribes komarovii* Pojark.
	subg. *Berisia*	*Ribes maximowiczianum* Kom.
	subg. *Grossularia*	*Ribes burejense* F.Schmidt
	subg. *Hemibotrya*	*Ribes fasciculatum* Siebold & Zucc.
	subg. *Ribesia*	*Ribes latifolium* Jancz.
	subg. *Ribesia*	*Ribes mandshuricum* (Maxim.) Kom.
	subg. *Ribesia*	*Ribes triste* Pall.
	subg. *Grossularioides*	*Ribes horridum* Rupr. ex Maxim.
North Korea	subg. *Berisia*	*Ribes komarovii* Pojark.
	subg. *Berisia*	*Ribes maximowiczianum* Kom.
	subg. *Grossularia*	*Ribes burejense* F.Schmidt
	subg. *Hemibotrya*	*Ribes fasciculatum* Siebold & Zucc.
	subg. *Ribesia*	*Ribes latifolium* Jancz.
	subg. *Ribesia*	*Ribes mandshuricum* (Maxim.) Kom.
	subg. *Ribesia*	*Ribes triste* Pall.
	subg. *Grossularioides*	*Ribes horridum* Rupr. ex Maxim.

**Table 3:** Common and endemic species in various countries and regions of Northeast Asia. The number ’1’ indicates species presence in each country/region and ‘0’ indicates absence in each country/region, and endemic species are marked as present ’1’ in only one country/region.

Species	Northeast Asia Region of Russia	Northeast China	Japan	Mongolia	South Korea	North Korea
*Ribes sachalinense* (F.Schmidt) Nakai	1	0	1	0	0	0
*Ribes altissimum* Turcz. ex Pojark.	1	1	0	1	0	0
*Ribes atropurpureum* C.A.Mey.	1	0	0	1	0	0
*Ribes diacantha* Pall.	1	1	0	1	1	0
*Ribes dikuscha* Fisch. ex Turcz.	1	0	0	0	0	0
*Ribes fontaneum* Bochkarn.	1	0	0	0	0	0
*Ribes frangrans* Pallas.	1	1	0	1	0	0
*Ribes graveolens* Bunge	1	0	0	1	0	0
*Ribes horridum* Rupr. ex Maxim.	1	1	1	0	1	1
*Ribes komarovii* Pojark.	1	1	0	0	1	1
*Ribes latifolium* Jancz.	1	1	1	0	1	1
*Ribes mandshuricum* (Maxim.) Kom.	1	1	0	0	1	1
*Ribes maximowiczianum* Kom.	1	1	1	0	1	1
*Ribes meyeri* Maxim.	1	0	0	1	0	0
*Ribes nigrum* L.	1	1	1	1	0	0
*Ribes palczewskii* (Jancz.) Pojark.	1	1	0	0	0	0
*Ribes pallidiflorum* Pojark.	1	0	0	0	0	0
*Ribes procumbens* Pall.	1	1	1	1	0	0
*Ribes pulchellum* Turcz.	1	1	0	1	0	0
*Ribes saxatile* Pall.	1	0	0	0	0	0
*Ribes triste* Pall.	1	1	1	0	1	1
*Ribes burejense* F.Schmidt	0	1	0	0	1	1
*Ribes fasciculatum* var. *chinense* Maxim.	0	1	0	0	0	0
*Ribes giraldii* Jancz.	0	1	0	0	0	0
*Ribes ambiguum* Maxim.	0	0	1	0	0	0
*Ribes fasciculatum* Siebold & Zucc.	0	0	1	0	1	1
*Ribes fujisanense* S.Sakag. & M.Oishi	0	0	1	0	0	0
*Ribes japonicum* Maxim.	0	0	1	0	0	0
*Ribes sinanense* F.Maek.	0	0	1	0	0	0
*Ribes heterotrichum* C.A.Mey.	0	0	0	1	0	0

**Table 4:** Explanatory variables used to model *Ribes* L. species richness in Northeast Asia.

Variable Type	Variable	Variable Description	[Max, Min]	Mean Value	Unit	T-Value Correlation Coefficient	*p*-Value
Climate	BIO1	Annual Mean Temperature	[−17.101, 21.843]	−1.906 ± 7.515	°C	11.81	<2 × 10^−16^ ***
	BIO2	Mean Diurnal Range	[1.628, 18.229]	10.795 ± 2.523	-	7.039	2.01 × 10^−12^ ***
	BIO3	Isothermality	[12.878, 80.903]	23.246 ± 5.584	-	13.59	<2 × 10^−16^ ***
	BIO4	Temperature Seasonality	[212.407, 2310.871]	1364.185 ± 347.707	-	−9.213	<2 × 10^−16^ ***
	BIO5	Max Temperature of Warmest Month	[−0.315, 37.688]	22.19 ± 5.379	°C	7.195	6.52 × 10^−13^ ***
	BIO6	Min Temperature of Coldest Month	[−48.566, 10.161]	−25.251 ± 10.684	°C	10.10	<2 × 10^−16^ ***
	BIO7	Temperature Annual Range	[6.886, 71.383]	47.441 ± 9.942	-	−6.805	<2 × 10^−16^ ***
	BIO8	Mean Temperature of Wettest Quarter	[−5.28, 28.312]	13.837 ± 5.506	°C	7.965	1.76 × 10^−15^ ***
	BIO9	Mean Temperature of Driest Quarter	[−38.054, 22.096]	−14.75 ± 10.108	°C	9.729	<2 × 10^−16^ ***
	BIO10	Mean Temperature of Warmest Quarter	[−2.344, 28.312]	14.643 ± 5.192	°C	8.744	<2 × 10^−16^ ***
	BIO11	Mean Temperature of Coldest Quarter	[−42.866, 14.825]	−18.707 ± 10.512	°C	11.39	<2 × 10^−16^ ***
	BIO12	Annual Precipitation	[13.653, 3238.4]	469.173 ± 275.078	mm	1.698	0.0896 ^ns^
	BIO13	Precipitation of Wettest Month	[4.592, 538]	82.711 ± 49.824	mm	8.924	<2 × 10^−16^ ***
	BIO14	Precipitation of Driest Month	[0, 138.8]	13.942 ± 12.228	mm	−1.224	0.221 ^ns^
	BIO15	Precipitation Seasonality	[15.385, 140.815]	62.705 ± 29.082		8.141	4.21 × 10^−16^ ***
	BIO16	Precipitation of Wettest Quarter	[11.122, 1274.2]	215.826 ± 125.478	mm	4.968	6.82 × 10^−7^ ***
	BIO17	Precipitation of Driest Quarter	[0, 470.6]	48.234 ± 40.823	mm	20.438	0.621 ^ns^
	BIO18	Precipitation of Warmest Quarter	[11.122, 964.6]	210.241 ± 116.819	mm	4.800	1.6 × 10^−6^ ***
	BIO19	Precipitation of Coldest Quarter	[0, 503.486]	57.579 ± 50.016	mm	14.012	<2 × 10^−16^ ***
Topography	ELE	Mean Elevation	[−29, 5936.245]	683.056 ± 974.05	m	8.502	0.00821 **

Note: *** *p* < 0.001; ** *p* < 0.01; ns, not significant.

## Data Availability

Data is contained within the article or supplementary material.

## Supplementary Materials

The following supporting information can be downloaded at: https://www.mdpi.com/article/10.3390/plants14121780/s1.
